# Bis(μ-3,5-dimethyl-4*H*-1,2,4-triazol-4-amine-κ^2^ *N* ^1^:*N* ^2^)bis­[bis­(thio­cyanato-κ*N*)zinc]–bis­(3,5-dimethyl-4*H*-1,2,4-triazol-4-amine-κ*N* ^1^)bis­(thio­cyanato-κ*N*)zinc (1/2)

**DOI:** 10.1107/S1600536811027887

**Published:** 2011-07-16

**Authors:** Hai-Yan Ge, Bao-Long Li

**Affiliations:** a College of Chemical Engineering, Taishan Medical College, Taian 271016, People’s Republic of China; b College of Chemistry, Chemical Engineering and Materials Science, The Key Laboratory of Organic Synthesis of Jiangsu Province, Soochow University, Suzhou 215123, People’s Republic of China

## Abstract

In the crystal structure of the title 1:2 adduct, [Zn_2_(NCS)_4_(C_4_H_8_N_4_)_2_]·2[Zn(NCS)_2_(C_4_H_8_N_4_)_2_] or (I*a*)·2(I*b*), each Zn^II^ atom is coordinated in a distorted tetra­hedral geometry by four N atoms from two triazole rings of two 4-amino-3,5-dimethyl-1,2,4-triazole (admt) ligands and two NCS^−^ ligands. In (I*a*), double *N* ^1^:*N* ^2^-bridging admt ligands connect two Zn^II^ atoms, forming a dimer with a Zn_2_(admt)_2_ six-membered metallacycle located on a crystallographic inversion center. In (I*b*), the admt ligands exhibit monodentate *N* ^1^-coordination modes. Weak N—H⋯N, N—H⋯S and C—H⋯S hydrogen bonds play an important role in the inter­molecular packing. The S and C atoms of two thiocyanato ligands are disordered over two sets of sites in ratios of 0.57 (3):0.43 (3) and 0.63 (3):0.37 (3), respectively.

## Related literature

For background to transition metal complexes of 1,2,4-triazole derivatives, see: Haasnoot (2000 ▶); Liu *et al.* (1999 ▶, 2003 ▶); Zhao *et al.* (2002 ▶); Yi *et al.* (2004 ▶); Lavrenova *et al.* (1992 ▶); Zhang *et al.* (2007 ▶, 2011 ▶). For related structures, see: Lavrenova *et al.* (1992 ▶); Zhang *et al.* (2007 ▶, 2011 ▶).
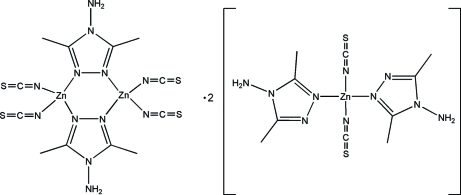


## Experimental

### 

#### Crystal data

[Zn_2_(NCS)_4_(C_4_H_8_N_4_)_2_]·2[Zn(NCS)_2_(C_4_H_8_N_4_)_2_]
*M* *_r_* = 1399.08
Triclinic, 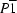

*a* = 8.7665 (8) Å
*b* = 9.3100 (5) Å
*c* = 20.661 (3) Å
α = 92.560 (9)°
β = 95.926 (2)°
γ = 115.427 (6)°
*V* = 1507.3 (3) Å^3^
*Z* = 1
Mo *K*α radiation
μ = 1.91 mm^−1^
*T* = 223 K
0.34 × 0.30 × 0.14 mm

#### Data collection

Rigaku Mercury CCD diffractometer
Absorption correction: multi-scan (*REQAB*; Jacobson, 1998 ▶) *T*_min_ = 0.564, *T*_max_ = 0.776
14831 measured reflections
5484 independent reflections
4550 reflections with *I* > 2σ(*I*)
*R*_int_ = 0.034

#### Refinement

*R*[*F* ^2^ > 2σ(*F* ^2^)] = 0.042
*wR*(*F* ^2^) = 0.090
*S* = 1.04
5484 reflections
387 parameters
30 restraints
H atoms treated by a mixture of independent and constrained refinement
Δρ_max_ = 0.55 e Å^−3^
Δρ_min_ = −0.36 e Å^−3^

### 

Data collection: *CrystalClear* (Rigaku, 2000 ▶); cell refinement: *CrystalClear*; data reduction: *CrystalClear*; program(s) used to solve structure: *SHELXS97* (Sheldrick, 2008 ▶); program(s) used to refine structure: *SHELXL97* (Sheldrick, 2008 ▶); molecular graphics: *SHELXTL* (Sheldrick, 2008 ▶); software used to prepare material for publication: *SHELXTL*.

## Supplementary Material

Crystal structure: contains datablock(s) I, global. DOI: 10.1107/S1600536811027887/zq2110sup1.cif

Structure factors: contains datablock(s) I. DOI: 10.1107/S1600536811027887/zq2110Isup2.hkl

Additional supplementary materials:  crystallographic information; 3D view; checkCIF report

## Figures and Tables

**Table 1:** Selected bond lengths (Å)

Zn1—N5	1.914 (3)
Zn1—N6	1.954 (3)
Zn1—N2^i^	2.011 (3)
Zn1—N1	2.012 (3)
Zn2—N16	1.926 (3)
Zn2—N15	1.962 (3)
Zn2—N7	1.990 (3)
Zn2—N11	2.000 (3)

**Table 2:** Hydrogen-bond geometry (Å, °)

*D*—H⋯*A*	*D*—H	H⋯*A*	*D*⋯*A*	*D*—H⋯*A*
N4—H*W*1⋯S2^ii^	0.89 (2)	2.83 (3)	3.495 (3)	133 (3)
N4—H*W*2⋯N12	0.88 (2)	2.31 (2)	3.157 (4)	162 (3)
N10—H*W*3⋯N12^iii^	0.85 (2)	2.47 (2)	3.251 (4)	152 (3)
N10—H*W*4⋯S4^iii^	0.87 (2)	2.80 (2)	3.641 (4)	163 (3)
N14—H*W*5⋯N8^iv^	0.88 (2)	2.20 (2)	3.061 (4)	167 (3)
N14—H*W*6⋯S3*A*^v^	0.87 (2)	2.83 (3)	3.617 (9)	151 (3)
N14—H*W*6⋯S3*B*^v^	0.87 (2)	2.83 (3)	3.542 (18)	140 (3)
C13—H13*B*⋯S3*A*^v^	0.97	2.82	3.458 (7)	124

Symmetry codes: (ii) 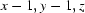; (iii) 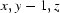; (iv) 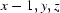; (v) 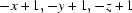.

**Fig. 1.**
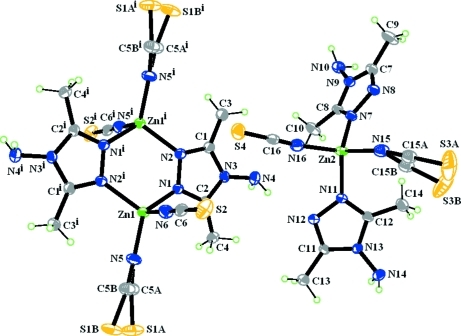
The molecular structure of the title adduct. Displacement ellipsoids are drawn at the 30% probability level. H atoms are presented as a small spheres of arbitrary radius. Symmetry code (i): -x+1, -y+1, -z.

**Fig. 2.**
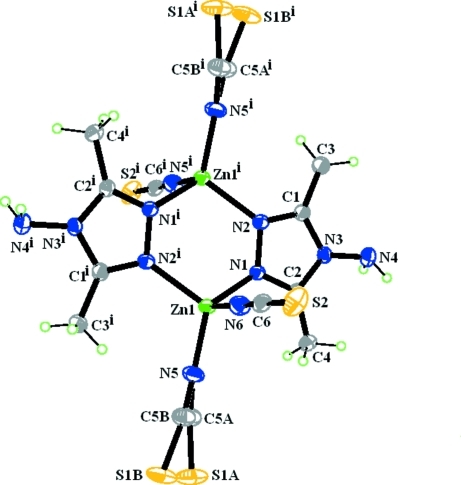
The dimeric structure of (Ia) with displacement ellipsoids drawn at the 30% probability level. Symmetry code (i): -x+1, -y+1, -z.

**Fig. 3.**
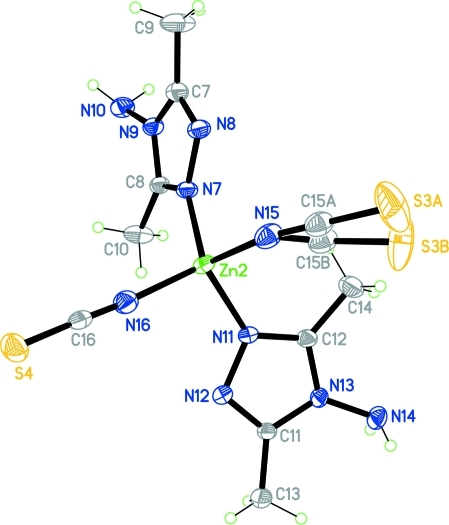
The mononuclear structure of (Ib) with displacement ellipsoids drawn at the 30% probability level.

**Fig. 4.**
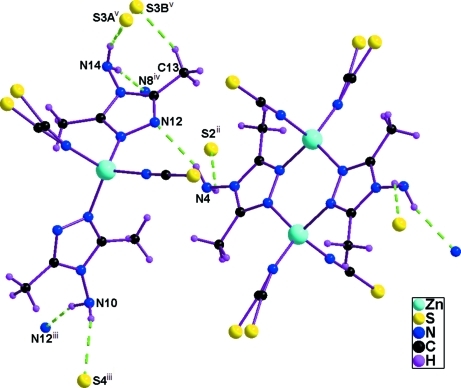
A view of the hydrogen bond interactions (dashed lines) in the crystal structure pf the title compound. Symmetry codes: (ii) x-1, y-1, z; (iii) x, y-1, z; (iv) x-1, y, z; (v) -x+1, -y+1, -z+1.

## supplementary crystallographic information

### Comment

A large number of mononuclear, oligonuclear and polynuclear transition metal complexes of 1,2,4-triazole derivatives have been synthesized and characterized due to their magnetic properties and novel topologies (Haasnoot, 2000). For 4-amino-3,5-dimethyl-1,2,4-triazole (admt), several Mn^II^ (Liu *et al.*, 1999), Co^II^, Ni^II^ (Zhao *et al.*, 2002), Cu^II^ (Liu *et al.*, 2003) and Cd^II^ compounds (Yi *et al.*, 2004) were synthesized. Two Zn^II^-admt compounds [Zn_2_(admt)_2_Cl_4_] (Lavrenova *et al.*, 1992) and [Zn_2_(admt)_2_I_4_] (Zhang *et al.*, 2011) were previously synthesized. Here we report the preparation and crystal structure of the title Zn^II ^adduct [Zn_2_(admt)_2_(NCS)_4_].2[Zn(admt)_2_(NCS)_2_] (I), containing a dimer [Zn_2_(admt)_2_(NCS)_4_] (I*a*) and two mononuclear [Zn(admt)_2_(NCS)_2_] (I*b*) species in the same crystal structure (Fig. 1).

The molecular structure of the neutral dimer (I*a*) is similar to [Zn_2_(admt)_2_Cl_4_] (Lavrenova, *et al.*, 1992) and [Zn_2_(admt)_2_I_4_] (Zhang *et al.*, 2011). Two Zn^II^ centers are connected by two admt ligands, resulting in a discrete Zn_2_(admt)_2_ six-membered metallacycle (geometric center located on a crystallographic inversion center) which represents the smallest closed cyclic structure with a 1:1 metal-to-ligand ratio (Fig. 2). One thiocyanate ligand is partially disordered over two sets of positions with site-occupancy factors of 0.57 (3) and 0.43 (3). Each Zn^II^ center is four-coordinated by two nitrogen donors of two admt ligands with Zn1—N1 = 2.012 (3) Å and Zn1—N2^i^ = 2.011 (3) Å (symmetry code i: -*x* + 1,-*y* + 1,-*z*) and two nitrogen atoms of two thyocyanate ligands with Zn1—N5 = 1.914 (3) Å and Zn1—N6 = 1.954 (3) Å, forming a distorted tetrahedral geometry.

In the molecular structure of (I*b*), a mononuclear species, the Zn^II^ atom is also four-coordinated by two nitrogen donors of two admt ligands with Zn2—N7 = 1.990 (3) Å and Zn2—N11 = 2.000 (3) Å and two nitrogen atoms of two thyocyante ligands with Zn2—N15 = 1.962 (3) Å and Zn2—N16 = 1.926 (3)Å, forming a distorted tetrahedral geometry. One thiocyanate ligand is also partially disordered over two sets of positions with site-occupancy factors of 0.63 (3) and 0.37 (3).

The Zn—N (triazole) and Zn—N (NCS) bond lengths in the dimer (I*a*) are similar to the values in the mononuclear (I*b*) and other Zn-triazole complexes (Zhang, *et al.*, 2007 and 2011; Lavrenova, *et al.*, 1992). The N—Zn—N bond angles in (I*a*) are in the range of 103.49 (12) to 115.06 (13)°, and in the range of 103.14 (13) to 115.73 (12)° in (I*b*). In comparison, the Zn/admt ratio is 1:1 in (I*a*), but 1:2 in (I*b*). The ligand admt, a 4-substituted 1,2,4-triazole, exhibits a *N^1^*,*N^2^*-bidentate bridging coordination mode in (I*a*), two admt ligands bridging two Zn^II^ atoms to form a dimer with a Zn···Zn distance of 3.6708 (8) Å. However, in the mononuclear species (I*b*), admt shows a *N^1^* monodentate coordination mode. For a 4-substituted 1,2,4-triazole, blocking the N4 donor position through substitution, only the *N^1^* monodentate and *N^1^*,*N^2^*-bidentate coordination modes are possible.

In the crystal structure of the title compound, there are hydrogen bonding interactions between amino groups NH_2_ and S atoms [N4···S2^ii^ (ii = *x*-1, *y*-1, *z*) = 3.495 (4) Å; N10···S4^iii^ (iii = *x*, *y*-1, *z*) = 3.641 (4) Å; N14···S3A^v^ (v = -*x*+1, -*y*+1, -*z*+1) = 3.617 (9) Å; N14···S3B^v ^= 3.542 (18) Å], and between amino groups NH_2_ and the 2-position triazole N atom of (I*b*) [N4···N12 = 3.157 (4) Å; N10···N12^iii^ = 3.251 (4) Å; N14···N8^iv^ (iv = *x*-1, *y*, *z*) = 3.061 (4) Å]. There are also weak hydrogen bonding interactions between methyl hydrogen atoms and S atoms [C13···S3A^v^ = 3.458 (7) Å]. No obviously π-π stacking interactions between the triazole rings were observed. These hydrogen bonding interaction stabilize the crystal structure of the title adduct (Fig. 3).

### Experimental

A 15 ml aqueaous solution of 4-amino-3,5-dimethyl-1,2,4-triazole (admt) (1.0 mmol) was added to 10 ml aqueous solution Zn(NO_3_)_2_^.^6H_2_O (1.0 mmol) and KSCN (2.0 mmol) with stirring. The result solution was placed at room temperature. Colourless crystals were obtained after about one month. Anal. Calcd. for C_32_H_48_N_32_S_8_Zn_4_: C, 27.47; H, 3.46; N, 32.05%. Found: C, 27.39; H, 3.41; N, 31.87%.

### Refinement

The methyl H atoms were placed in idealized positions and refined as riding, with C—H distances of 0.97Å and *U*_iso_(H) = 1.5*U*_eq_(C). H atoms bonded to N atoms were located in a difference map and refined with distance restraints of N—H = 0.87 (2) Å, and with *U*_iso_(H) = 1.2*U*_eq_(N).

### Crystal data

**Table:**

[Zn_2_(NCS)_4_(C_4_H_8_N_4_)_2_]·2[Zn(NCS)_2_(C_4_H_8_N_4_)_2_]	*Z* = 1
*M**_r_* = 1399.08	*F*(000) = 712
Triclinic, *P*1	*D*_x_ = 1.541 Mg m^−^^3^
Hall symbol: -P 1	Mo *K*α radiation, λ = 0.71070 Å
*a* = 8.7665 (8) Å	Cell parameters from 5140 reflections
*b* = 9.3100 (5) Å	θ = 3.1–25.4°
*c* = 20.661 (3) Å	µ = 1.91 mm^−^^1^
α = 92.560 (9)°	*T* = 223 K
β = 95.926 (2)°	Block, colourless
γ = 115.427 (6)°	0.34 × 0.30 × 0.14 mm
*V* = 1507.3 (3) Å^3^	

### Data collection

**Table:**

Rigaku Mercury CCD diffractometer	5484 independent reflections
Radiation source: fine-focus sealed tube	4550 reflections with *I* > 2σ(*I*)
graphite	*R*_int_ = 0.034
Detector resolution: 7.31 pixels mm^-1^	θ_max_ = 25.4°, θ_min_ = 3.1°
ω scans	*h* = −10→10
Absorption correction: multi-scan (REQAB; Jacobson, 1998)	*k* = −11→11
*T*_min_ = 0.564, *T*_max_ = 0.776	*l* = −22→24
14831 measured reflections	

### Refinement

**Table:**

Refinement on *F*^2^	Primary atom site location: structure-invariant direct methods
Least-squares matrix: full	Secondary atom site location: difference Fourier map
*R*[*F*^2^ > 2σ(*F*^2^)] = 0.042	Hydrogen site location: inferred from neighbouring sites
*wR*(*F*^2^) = 0.090	H atoms treated by a mixture of independent and constrained refinement
*S* = 1.04	*w* = 1/[σ^2^(*F*_o_^2^) + (0.0309*P*)^2^ + 1.5778*P*] where *P* = (*F*_o_^2^ + 2*F*_c_^2^)/3
5484 reflections	(Δ/σ)_max_ < 0.001
387 parameters	Δρ_max_ = 0.55 e Å^−^^3^
30 restraints	Δρ_min_ = −0.36 e Å^−^^3^

### Special details

**Table:**

Geometry. All e.s.d.'s (except the e.s.d. in the dihedral angle between two l.s. planes) are estimated using the full covariance matrix. The cell e.s.d.'s are taken into account individually in the estimation of e.s.d.'s in distances, angles and torsion angles; correlations between e.s.d.'s in cell parameters are only used when they are defined by crystal symmetry. An approximate (isotropic) treatment of cell e.s.d.'s is used for estimating e.s.d.'s involving l.s. planes.
Refinement. Refinement of *F*^2^ against ALL reflections. The weighted *R*-factor *wR* and goodness of fit *S* are based on *F*^2^, conventional *R*-factors *R* are based on *F*, with *F* set to zero for negative *F*^2^. The threshold expression of *F*^2^ > σ(*F*^2^) is used only for calculating *R*-factors(gt) *etc*. and is not relevant to the choice of reflections for refinement. *R*-factors based on *F*^2^ are statistically about twice as large as those based on *F*, and *R*- factors based on ALL data will be even larger.

### Fractional atomic coordinates and isotropic or equivalent isotropic displacement parameters (Å^2^)

**Table:**

	*x*	*y*	*z*	*U*_iso_*/*U*_eq_	Occ. (<1)
Zn1	0.41245 (5)	0.63167 (5)	0.031974 (19)	0.03212 (12)	
Zn2	0.54622 (5)	0.17586 (5)	0.34171 (2)	0.03310 (12)	
S1A	−0.0157 (7)	0.7863 (17)	0.0518 (4)	0.065 (2)	0.57 (3)
C5A	0.1191 (16)	0.7222 (15)	0.0350 (8)	0.0438 (11)	0.57 (3)
S1B	0.032 (2)	0.858 (2)	0.0278 (11)	0.087 (5)	0.43 (3)
C5B	0.143 (2)	0.7546 (19)	0.0296 (10)	0.0438 (11)	0.43 (3)
S3A	0.786 (2)	0.3793 (9)	0.5571 (3)	0.110 (3)	0.63 (3)
C15A	0.7285 (16)	0.3210 (15)	0.4808 (4)	0.0438 (11)	0.63 (3)
S3B	0.717 (2)	0.397 (2)	0.5555 (7)	0.111 (6)	0.37 (3)
C15B	0.690 (3)	0.329 (3)	0.4793 (7)	0.0438 (11)	0.37 (3)
S2	0.82933 (14)	0.97029 (14)	0.20364 (5)	0.0594 (3)	
S4	0.80473 (14)	0.52143 (13)	0.18706 (6)	0.0549 (3)	
N1	0.3658 (3)	0.4141 (3)	0.06001 (13)	0.0294 (6)	
N2	0.4699 (3)	0.3396 (3)	0.04834 (13)	0.0318 (6)	
N3	0.3122 (3)	0.2214 (3)	0.12156 (13)	0.0305 (6)	
N4	0.2470 (4)	0.1121 (4)	0.16740 (15)	0.0408 (7)	
N5	0.2201 (4)	0.6795 (4)	0.02509 (17)	0.0471 (8)	
N6	0.5860 (4)	0.7776 (4)	0.10139 (16)	0.0439 (8)	
N7	0.5476 (3)	−0.0370 (3)	0.33052 (14)	0.0323 (7)	
N8	0.6787 (4)	−0.0591 (4)	0.36615 (14)	0.0384 (7)	
N9	0.5265 (4)	−0.2668 (3)	0.29609 (14)	0.0329 (7)	
N10	0.4718 (4)	−0.4100 (4)	0.25494 (17)	0.0421 (8)	
N11	0.3205 (3)	0.1727 (3)	0.35209 (13)	0.0327 (7)	
N12	0.2521 (4)	0.2503 (3)	0.31036 (13)	0.0343 (7)	
N13	0.1194 (3)	0.1971 (3)	0.39608 (13)	0.0292 (6)	
N14	0.0123 (4)	0.1907 (4)	0.44340 (15)	0.0401 (7)	
N15	0.6844 (4)	0.2796 (4)	0.42611 (16)	0.0474 (8)	
N16	0.6258 (4)	0.3019 (4)	0.27023 (16)	0.0446 (8)	
C1	0.4354 (4)	0.2233 (4)	0.08645 (16)	0.0326 (8)	
C2	0.2705 (4)	0.3398 (4)	0.10392 (15)	0.0286 (7)	
C3	0.5138 (5)	0.1114 (4)	0.09104 (19)	0.0443 (9)	
H3A	0.4279	0.0037	0.0764	0.066*	
H3B	0.5608	0.1156	0.1361	0.066*	
H3C	0.6041	0.1413	0.0637	0.066*	
C4	0.1382 (5)	0.3744 (5)	0.12993 (18)	0.0405 (9)	
H4C	0.1100	0.4433	0.1022	0.061*	
H4D	0.1804	0.4274	0.1739	0.061*	
H4E	0.0371	0.2752	0.1308	0.061*	
C6	0.6884 (5)	0.8569 (4)	0.14333 (19)	0.0374 (8)	
C7	0.6631 (5)	−0.1990 (4)	0.34402 (18)	0.0395 (9)	
C8	0.4587 (4)	−0.1629 (4)	0.28850 (16)	0.0312 (8)	
C9	0.7755 (6)	−0.2747 (6)	0.3658 (2)	0.0694 (14)	
H9A	0.8615	−0.2061	0.4012	0.104*	
H9B	0.8305	−0.2903	0.3295	0.104*	
H9C	0.7079	−0.3773	0.3810	0.104*	
C10	0.3097 (5)	−0.1893 (5)	0.2399 (2)	0.0510 (11)	
H10C	0.2944	−0.0922	0.2395	0.077*	
H10D	0.2083	−0.2753	0.2516	0.077*	
H10E	0.3293	−0.2177	0.1968	0.077*	
C11	0.1301 (4)	0.2624 (4)	0.33821 (16)	0.0286 (7)	
C12	0.2390 (4)	0.1428 (4)	0.40368 (17)	0.0317 (8)	
C13	0.0177 (5)	0.3329 (5)	0.31128 (17)	0.0406 (9)	
H13A	0.0787	0.4160	0.2843	0.061*	
H13B	−0.0168	0.3785	0.3469	0.061*	
H13C	−0.0825	0.2505	0.2850	0.061*	
C14	0.2714 (5)	0.0639 (5)	0.4602 (2)	0.0513 (11)	
H14C	0.3331	0.0038	0.4482	0.077*	
H14D	0.1638	−0.0082	0.4734	0.077*	
H14E	0.3388	0.1440	0.4962	0.077*	
C16	0.7019 (4)	0.3929 (4)	0.23544 (18)	0.0351 (8)	
HW1	0.139 (3)	0.046 (4)	0.1524 (17)	0.042*	
HW2	0.238 (5)	0.164 (4)	0.2027 (13)	0.042*	
HW3	0.441 (5)	−0.481 (4)	0.2816 (15)	0.042*	
HW4	0.558 (3)	−0.405 (4)	0.2359 (17)	0.042*	
HW5	−0.091 (3)	0.127 (4)	0.4243 (17)	0.042*	
HW6	0.036 (5)	0.290 (3)	0.4554 (18)	0.042*	

### Atomic displacement parameters (Å^2^)

**Table:**

	*U*^11^	*U*^22^	*U*^33^	*U*^12^	*U*^13^	*U*^23^
Zn1	0.0362 (2)	0.0312 (2)	0.0369 (2)	0.02067 (19)	0.01070 (18)	0.00640 (17)
Zn2	0.0314 (2)	0.0340 (2)	0.0403 (2)	0.01972 (19)	0.00779 (18)	0.00290 (18)
S1A	0.0510 (18)	0.081 (4)	0.077 (3)	0.047 (2)	−0.0059 (19)	−0.026 (3)
C5A	0.034 (3)	0.041 (2)	0.055 (2)	0.017 (2)	0.0000 (18)	−0.0045 (19)
S1B	0.078 (5)	0.085 (7)	0.111 (8)	0.067 (6)	−0.048 (6)	−0.056 (6)
C5B	0.034 (3)	0.041 (2)	0.055 (2)	0.017 (2)	0.0000 (18)	−0.0045 (19)
S3A	0.182 (8)	0.066 (2)	0.046 (2)	0.030 (4)	−0.021 (3)	−0.0143 (17)
C15A	0.034 (3)	0.041 (2)	0.055 (2)	0.017 (2)	0.0000 (18)	−0.0045 (19)
S3B	0.116 (7)	0.085 (7)	0.067 (5)	−0.018 (4)	0.038 (4)	−0.033 (4)
C15B	0.034 (3)	0.041 (2)	0.055 (2)	0.017 (2)	0.0000 (18)	−0.0045 (19)
S2	0.0541 (6)	0.0548 (7)	0.0472 (6)	0.0039 (5)	0.0093 (5)	−0.0087 (5)
S4	0.0566 (7)	0.0515 (6)	0.0570 (7)	0.0211 (5)	0.0149 (5)	0.0211 (5)
N1	0.0330 (15)	0.0278 (15)	0.0312 (15)	0.0160 (13)	0.0072 (12)	0.0035 (12)
N2	0.0374 (16)	0.0299 (15)	0.0337 (16)	0.0195 (13)	0.0074 (13)	0.0031 (13)
N3	0.0359 (16)	0.0276 (15)	0.0283 (15)	0.0133 (13)	0.0062 (13)	0.0065 (12)
N4	0.0488 (19)	0.0378 (18)	0.0341 (18)	0.0158 (16)	0.0101 (16)	0.0085 (14)
N5	0.0395 (18)	0.051 (2)	0.063 (2)	0.0297 (17)	0.0105 (16)	0.0110 (17)
N6	0.0486 (19)	0.0391 (18)	0.0459 (19)	0.0225 (16)	0.0027 (17)	−0.0032 (16)
N7	0.0350 (16)	0.0318 (16)	0.0379 (17)	0.0216 (14)	0.0051 (13)	0.0070 (13)
N8	0.0424 (17)	0.0438 (18)	0.0373 (17)	0.0281 (15)	0.0006 (14)	−0.0003 (14)
N9	0.0368 (16)	0.0293 (15)	0.0369 (16)	0.0184 (13)	0.0055 (14)	0.0043 (13)
N10	0.047 (2)	0.0292 (17)	0.054 (2)	0.0193 (16)	0.0073 (16)	0.0054 (15)
N11	0.0351 (16)	0.0395 (17)	0.0342 (16)	0.0253 (14)	0.0075 (13)	0.0078 (13)
N12	0.0366 (16)	0.0415 (17)	0.0335 (16)	0.0243 (14)	0.0082 (13)	0.0059 (13)
N13	0.0236 (14)	0.0370 (16)	0.0296 (15)	0.0140 (13)	0.0094 (12)	0.0067 (12)
N14	0.0337 (17)	0.052 (2)	0.0390 (18)	0.0202 (17)	0.0140 (14)	0.0058 (16)
N15	0.0370 (17)	0.054 (2)	0.052 (2)	0.0229 (16)	−0.0020 (16)	−0.0105 (17)
N16	0.0428 (18)	0.0456 (19)	0.053 (2)	0.0238 (16)	0.0151 (16)	0.0129 (17)
C1	0.040 (2)	0.0259 (17)	0.0331 (19)	0.0150 (16)	0.0073 (16)	0.0042 (15)
C2	0.0316 (18)	0.0267 (17)	0.0248 (17)	0.0108 (15)	0.0025 (14)	−0.0006 (14)
C3	0.064 (3)	0.038 (2)	0.044 (2)	0.032 (2)	0.017 (2)	0.0111 (17)
C4	0.042 (2)	0.046 (2)	0.041 (2)	0.0237 (18)	0.0148 (17)	0.0091 (17)
C6	0.043 (2)	0.0328 (19)	0.041 (2)	0.0175 (18)	0.0173 (19)	0.0079 (17)
C7	0.044 (2)	0.042 (2)	0.040 (2)	0.0264 (19)	0.0041 (18)	0.0049 (17)
C8	0.0326 (18)	0.0298 (18)	0.0367 (19)	0.0173 (16)	0.0095 (15)	0.0084 (16)
C9	0.075 (3)	0.070 (3)	0.079 (3)	0.056 (3)	−0.023 (3)	−0.012 (3)
C10	0.048 (2)	0.037 (2)	0.069 (3)	0.0246 (19)	−0.011 (2)	0.003 (2)
C11	0.0298 (17)	0.0305 (18)	0.0291 (18)	0.0173 (15)	0.0013 (14)	0.0021 (14)
C12	0.0267 (17)	0.0367 (19)	0.037 (2)	0.0172 (16)	0.0074 (15)	0.0097 (16)
C13	0.043 (2)	0.054 (2)	0.035 (2)	0.0311 (19)	0.0023 (17)	0.0040 (17)
C14	0.048 (2)	0.065 (3)	0.053 (3)	0.033 (2)	0.015 (2)	0.029 (2)
C16	0.0323 (19)	0.037 (2)	0.042 (2)	0.0225 (17)	0.0021 (17)	−0.0013 (17)

### Geometric parameters (Å, °)

**Table:**

Zn1—N5	1.914 (3)	N10—HW4	0.868 (18)
Zn1—N6	1.954 (3)	N11—C12	1.318 (4)
Zn1—N2^i^	2.011 (3)	N11—N12	1.395 (4)
Zn1—N1	2.012 (3)	N12—C11	1.309 (4)
Zn2—N16	1.926 (3)	N13—C12	1.344 (4)
Zn2—N15	1.962 (3)	N13—C11	1.359 (4)
Zn2—N7	1.990 (3)	N13—N14	1.410 (4)
Zn2—N11	2.000 (3)	N14—HW5	0.879 (18)
S1A—C5A	1.595 (10)	N14—HW6	0.871 (18)
C5A—N5	1.147 (10)	N16—C16	1.161 (4)
S1B—C5B	1.629 (12)	C1—C3	1.475 (5)
C5B—N5	1.173 (12)	C2—C4	1.474 (5)
S3A—C15A	1.600 (9)	C3—H3A	0.9700
C15A—N15	1.147 (8)	C3—H3B	0.9700
S3B—C15B	1.626 (13)	C3—H3C	0.9700
C15B—N15	1.158 (12)	C4—H4C	0.9700
S2—C6	1.624 (4)	C4—H4D	0.9700
S4—C16	1.615 (4)	C4—H4E	0.9700
N1—C2	1.310 (4)	C7—C9	1.485 (5)
N1—N2	1.395 (4)	C8—C10	1.485 (5)
N2—C1	1.315 (4)	C9—H9A	0.9700
N2—Zn1^i^	2.011 (3)	C9—H9B	0.9700
N3—C2	1.355 (4)	C9—H9C	0.9700
N3—C1	1.357 (4)	C10—H10C	0.9700
N3—N4	1.399 (4)	C10—H10D	0.9700
N4—HW1	0.889 (18)	C10—H10E	0.9700
N4—HW2	0.882 (18)	C11—C13	1.478 (5)
N6—C6	1.147 (5)	C12—C14	1.474 (5)
N7—C8	1.312 (4)	C13—H13A	0.9700
N7—N8	1.395 (4)	C13—H13B	0.9700
N8—C7	1.305 (4)	C13—H13C	0.9700
N9—C8	1.342 (4)	C14—H14C	0.9700
N9—C7	1.365 (5)	C14—H14D	0.9700
N9—N10	1.413 (4)	C14—H14E	0.9700
N10—HW3	0.853 (18)		
			
N5—Zn1—N6	109.04 (14)	N2—C1—C3	127.0 (3)
N5—Zn1—N2^i^	115.00 (13)	N3—C1—C3	124.7 (3)
N6—Zn1—N2^i^	105.22 (12)	N1—C2—N3	108.2 (3)
N5—Zn1—N1	114.77 (12)	N1—C2—C4	127.1 (3)
N6—Zn1—N1	103.49 (12)	N3—C2—C4	124.7 (3)
N2^i^—Zn1—N1	108.28 (11)	C1—C3—H3A	109.5
N16—Zn2—N15	112.81 (14)	C1—C3—H3B	109.5
N16—Zn2—N7	112.84 (13)	H3A—C3—H3B	109.5
N15—Zn2—N7	106.15 (13)	C1—C3—H3C	109.5
N16—Zn2—N11	105.92 (12)	H3A—C3—H3C	109.5
N15—Zn2—N11	103.13 (12)	H3B—C3—H3C	109.5
N7—Zn2—N11	115.73 (11)	C2—C4—H4C	109.5
N5—C5A—S1A	177.3 (14)	C2—C4—H4D	109.5
N5—C5B—S1B	174.2 (18)	H4C—C4—H4D	109.5
N15—C15A—S3A	178.8 (7)	C2—C4—H4E	109.5
N15—C15B—S3B	174.4 (13)	H4C—C4—H4E	109.5
C2—N1—N2	108.0 (3)	H4D—C4—H4E	109.5
C2—N1—Zn1	127.6 (2)	N6—C6—S2	178.4 (4)
N2—N1—Zn1	122.2 (2)	N8—C7—N9	109.4 (3)
C1—N2—N1	107.5 (3)	N8—C7—C9	126.4 (4)
C1—N2—Zn1^i^	127.7 (2)	N9—C7—C9	124.2 (3)
N1—N2—Zn1^i^	122.1 (2)	N7—C8—N9	108.1 (3)
C2—N3—C1	108.1 (3)	N7—C8—C10	127.2 (3)
C2—N3—N4	129.1 (3)	N9—C8—C10	124.7 (3)
C1—N3—N4	122.8 (3)	C7—C9—H9A	109.5
N3—N4—HW1	108 (2)	C7—C9—H9B	109.5
N3—N4—HW2	109 (2)	H9A—C9—H9B	109.5
HW1—N4—HW2	103 (3)	C7—C9—H9C	109.5
C5A—N5—Zn1	164.9 (8)	H9A—C9—H9C	109.5
C5B—N5—Zn1	158.6 (10)	H9B—C9—H9C	109.5
C6—N6—Zn1	176.7 (3)	C8—C10—H10C	109.5
C8—N7—N8	108.9 (3)	C8—C10—H10D	109.5
C8—N7—Zn2	132.1 (2)	H10C—C10—H10D	109.5
N8—N7—Zn2	118.4 (2)	C8—C10—H10E	109.5
C7—N8—N7	106.2 (3)	H10C—C10—H10E	109.5
C8—N9—C7	107.5 (3)	H10D—C10—H10E	109.5
C8—N9—N10	123.4 (3)	N12—C11—N13	109.4 (3)
C7—N9—N10	128.7 (3)	N12—C11—C13	126.1 (3)
N9—N10—HW3	103 (3)	N13—C11—C13	124.5 (3)
N9—N10—HW4	108 (3)	N11—C12—N13	108.0 (3)
HW3—N10—HW4	114 (4)	N11—C12—C14	126.6 (3)
C12—N11—N12	108.5 (3)	N13—C12—C14	125.3 (3)
C12—N11—Zn2	128.6 (2)	C11—C13—H13A	109.5
N12—N11—Zn2	120.5 (2)	C11—C13—H13B	109.5
C11—N12—N11	106.4 (3)	H13A—C13—H13B	109.5
C12—N13—C11	107.6 (3)	C11—C13—H13C	109.5
C12—N13—N14	123.5 (3)	H13A—C13—H13C	109.5
C11—N13—N14	128.8 (3)	H13B—C13—H13C	109.5
N13—N14—HW5	105 (2)	C12—C14—H14C	109.5
N13—N14—HW6	106 (3)	C12—C14—H14D	109.5
HW5—N14—HW6	118 (4)	H14C—C14—H14D	109.5
C15A—N15—Zn2	163.6 (7)	C12—C14—H14E	109.5
C15B—N15—Zn2	148.3 (11)	H14C—C14—H14E	109.5
C16—N16—Zn2	167.0 (3)	H14D—C14—H14E	109.5
N2—C1—N3	108.2 (3)	N16—C16—S4	178.7 (3)
			
N5—Zn1—N1—C2	−39.3 (3)	N2—N1—C2—N3	1.0 (3)
N6—Zn1—N1—C2	79.4 (3)	Zn1—N1—C2—N3	−162.3 (2)
N2^i^—Zn1—N1—C2	−169.3 (3)	N2—N1—C2—C4	−178.2 (3)
N5—Zn1—N1—N2	159.6 (2)	Zn1—N1—C2—C4	18.5 (5)
N6—Zn1—N1—N2	−81.7 (2)	C1—N3—C2—N1	−0.8 (3)
N2^i^—Zn1—N1—N2	29.6 (3)	N4—N3—C2—N1	179.0 (3)
C2—N1—N2—C1	−0.8 (3)	C1—N3—C2—C4	178.5 (3)
Zn1—N1—N2—C1	163.5 (2)	N4—N3—C2—C4	−1.8 (5)
C2—N1—N2—Zn1^i^	162.0 (2)	N7—N8—C7—N9	−0.4 (4)
Zn1—N1—N2—Zn1^i^	−33.6 (3)	N7—N8—C7—C9	178.8 (4)
N16—Zn2—N7—C8	−66.9 (3)	C8—N9—C7—N8	0.8 (4)
N15—Zn2—N7—C8	169.0 (3)	N10—N9—C7—N8	173.8 (3)
N11—Zn2—N7—C8	55.3 (3)	C8—N9—C7—C9	−178.4 (4)
N16—Zn2—N7—N8	102.7 (2)	N10—N9—C7—C9	−5.4 (6)
N15—Zn2—N7—N8	−21.4 (3)	N8—N7—C8—N9	0.7 (4)
N11—Zn2—N7—N8	−135.1 (2)	Zn2—N7—C8—N9	171.0 (2)
C8—N7—N8—C7	−0.1 (4)	N8—N7—C8—C10	−178.8 (3)
Zn2—N7—N8—C7	−172.0 (2)	Zn2—N7—C8—C10	−8.4 (6)
N16—Zn2—N11—C12	−159.0 (3)	C7—N9—C8—N7	−0.9 (4)
N15—Zn2—N11—C12	−40.3 (3)	N10—N9—C8—N7	−174.3 (3)
N7—Zn2—N11—C12	75.2 (3)	C7—N9—C8—C10	178.5 (4)
N16—Zn2—N11—N12	1.3 (3)	N10—N9—C8—C10	5.1 (5)
N15—Zn2—N11—N12	120.0 (2)	N11—N12—C11—N13	0.5 (4)
N7—Zn2—N11—N12	−124.6 (2)	N11—N12—C11—C13	−178.4 (3)
C12—N11—N12—C11	−0.7 (4)	C12—N13—C11—N12	−0.2 (4)
Zn2—N11—N12—C11	−164.5 (2)	N14—N13—C11—N12	176.7 (3)
N15—Zn2—N16—C16	17.4 (14)	C12—N13—C11—C13	178.8 (3)
N7—Zn2—N16—C16	−102.9 (13)	N14—N13—C11—C13	−4.3 (5)
N11—Zn2—N16—C16	129.5 (13)	N12—N11—C12—N13	0.6 (4)
N1—N2—C1—N3	0.3 (3)	Zn2—N11—C12—N13	162.8 (2)
Zn1^i^—N2—C1—N3	−161.3 (2)	N12—N11—C12—C14	−179.4 (4)
N1—N2—C1—C3	179.9 (3)	Zn2—N11—C12—C14	−17.2 (5)
Zn1^i^—N2—C1—C3	18.3 (5)	C11—N13—C12—N11	−0.3 (4)
C2—N3—C1—N2	0.2 (4)	N14—N13—C12—N11	−177.4 (3)
N4—N3—C1—N2	−179.5 (3)	C11—N13—C12—C14	179.7 (4)
C2—N3—C1—C3	−179.3 (3)	N14—N13—C12—C14	2.6 (5)
N4—N3—C1—C3	0.9 (5)		

Symmetry codes: (i) −*x*+1, −*y*+1, −*z*.

### Hydrogen-bond geometry (Å, °)

**Table:**

*D*—H···*A*	*D*—H	H···*A*	*D*···*A*	*D*—H···*A*
N4—HW1···S2^ii^	0.89 (2)	2.83 (3)	3.495 (3)	133 (3)
N4—HW2···N12	0.88 (2)	2.31 (2)	3.157 (4)	162 (3)
N10—HW3···N12^iii^	0.85 (2)	2.47 (2)	3.251 (4)	152 (3)
N10—HW4···S4^iii^	0.87 (2)	2.80 (2)	3.641 (4)	163 (3)
N14—HW5···N8^iv^	0.88 (2)	2.20 (2)	3.061 (4)	167 (3)
N14—HW6···S3A^v^	0.87 (2)	2.83 (3)	3.617 (9)	151 (3)
N14—HW6···S3B^v^	0.87 (2)	2.83 (3)	3.542 (18)	140 (3)
C13—H13B···S3A^v^	0.97	2.82	3.458 (7)	124.

Symmetry codes: (ii) *x*−1, *y*−1, *z*; (iii) *x*, *y*−1, *z*; (iv) *x*−1, *y*, *z*; (v) −*x*+1, −*y*+1, −*z*+1.
